# Evaluation of a New Personalized Health Dashboard in Preventive Child Health Care: Protocol for a Mixed Methods Feasibility Randomized Controlled Trial

**DOI:** 10.2196/21942

**Published:** 2021-03-16

**Authors:** Miriam Weijers, Frans Feron, Jonne van der Zwet, Caroline Bastiaenen

**Affiliations:** 1 Department of Preventive Child Health Care Municipal Health Services South Limburg Heerlen Netherlands; 2 Department of Social Medicine Faculty of Health, Medicine and Life Sciences Care and Public Health Research Institute, Maastricht University Maastricht Netherlands; 3 Department of Epidemiology Faculty of Health, Medicine and Life Sciences Care and Public Health Research Institute, Maastricht University Maastricht Netherlands

**Keywords:** child health services, prevention, control, personalized health care, international classification of functioning, disability, health, patient, access to records, personalized, child, feasibility

## Abstract

**Background:**

A new dashboard, the 360ºCHILD-profile, was developed to adopt personalized health care within preventive child health care. On this profile, holistic health data are visualized in a single image to provide parents, adolescents, and caregivers direct access to a manageable résumé of a child’s medical record. Theoretical ordering, conforming to “International Classification of Functioning, Disability and Health for Children and Youth”, guides clinical reasoning toward the biopsychosocial concept of health. It is yet unknown if and how this promising tool functions in practice, and a variety of feasibility questions must be addressed.

**Objective:**

This paper describes the design and methods of a feasibility randomized controlled trial (RCT), with the aim of evaluating the RCT’s feasibility (recruitment, response, measure completion, and intervention allocation) and 360ºCHILD-profile’s feasibility (usability and potential effectiveness).

**Methods:**

A pragmatic mixed methods study design was chosen, starting with an RCT to measure feasibility and health literacy in 2 parallel groups (1:1). Qualitative research will then be used to understand and explain quantitative findings and to explore the stakeholders' perspectives on the potential of the 360ºCHILD-profile. Participants will include child health care professionals (n≥30), parents (n≥30), and caregivers (n≥10) of children who experience developmental problems (age 0-16 years). Children will only be able to participate if they are older than 11 years (adolescents, n≥10). The 2 groups included in the study will receive standard care. The experimental group will additionally receive personalized 360ºCHILD-profiles.

**Results:**

After an intervention period of 6 months, quantitative outcomes will be measured, analyzed (descriptive feasibility statistics and preliminary between-group differences) and used to purposively sample for semistructured interviews.

**Conclusions:**

Study results will provide knowledge for building theory on the 360ºCHILD-profile and designing future (effect) studies.

**Trial Registration:**

Netherlands Trial Register NTR6909; https://www.trialregister.nl/trial/6731

**International Registered Report Identifier (IRRID):**

DERR1-10.2196/21942

## Introduction

To more effectively address the increasing burden of preventable chronic diseases, it is a prerequisite for current reactive health care (treatment after a diagnosis) to make the transition to personalized health care (PHC) [1]. According to Snyderman [2], PHC stands for a lifetime, holistic approach of proactively offering predictive, preventive, personalized, and participatory care. The different PHC concepts can and must be introduced in practice as soon as possible, but it appears to be a challenging task to effectuate such a new approach within health care [2,3].

Dutch preventive child health care (CHC), as part of public health, offers a unique platform to adopt PHC in the short term. CHC proactively monitors children’s development and health to detect early deviance from normal variance and diseases or symptoms which cannot yet be clustered to a diagnosis. To fully apply PHC within the preventive CHC approach, a shift is needed toward prediction and prevention in the very early stages of disease progression when symptoms may not even be present [4,5]. To perform early detection and act upon disease progression is not an easy task because health processes are complex. The biopsychosocial model of health shows that health is a result of lifelong, multidimensional interactions between many biological–genetic characteristics and environmental factors. Therefore, to predict and protect health, insight into a broad set of health determinants is required. Critical to implementation of PHC is thus the availability and accessibility of high-quality, relevant lifetime health data.

From birth on, CHC collects health data about the child and environment, which are stored in an electronic medical dossier (EMD). However, accessibility of data is profoundly hindered due to the actual structure of EMDs, and thus support for the complex clinical reasoning process is insufficient [6,7]. It is not possible to generate an adequate overview of registered data in coherence with the relevant theoretical concepts (ie, the biopsychosocial model) [8]. As a result, much of the CHC-data that are highly relevant to understanding the complex processes underlying health are not available within the timeframe of CHC visits or other consultations with caregivers and parents.

**Figure 1:**
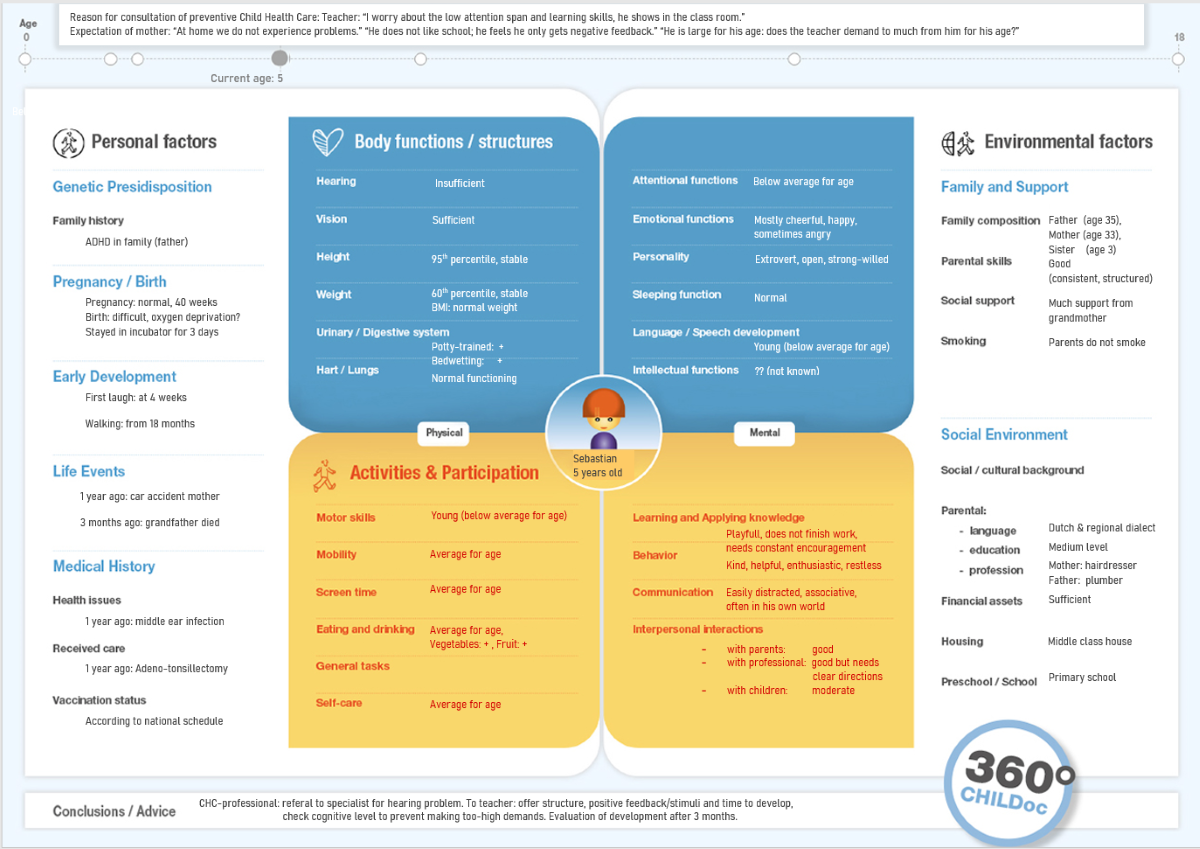
The 360°CHILD-profile: representation of a child’s health information available in the CHC electronic medical dossier, based on the "International Classification of Functioning, Disability and Health for Children and Youth” [10]. Child’s name, gender, and age appear in the center; the most recently acquired data about the child’s body functions and structures in physical and mental aspects appear in the blue area; and information on activities and participation appear in the yellow area. Data about genetic predisposition and history (medical, developmental, and life events) are listed in the left column, and information about environmental factors are in the right column. On top of the 360ºCHILD-profile, a timeline is provided with bullet points at which ages the child visited CHC, and a CHC and a 360ºCHILD-profile (snap-shot) is generated. At the bottom of the 360ºCHILD-profile, conclusions and advice from the most recent CHC visit are presented. CHC: child health care.

To address this problem, a 360ºCHILD-profile (Figure 1), which visualizes health information about the child and environment in a single digital image, has been originally developed and examined within daily practice of the Dutch CHC system [9].

Relevant CHC data, visualized on the 360ºCHILD-profile, are theoretically ordered according to the “International Classification of Functioning, Disability and Health for Children and Youth” (ICF-CY) [10]. The ICF-CY fits the CHC context and PHC concepts, as it is built on the integrated biopsychosocial approach of health and describes a broad variety of individual characteristics and environmental factors in concrete, neutral (if not positive) formulations [10]. The 360ºCHILD-profile was developed as a dashboard that provides a quick, systemic, and comprehensible representation of a child’s individually unique set of health determinants (protective and risk factors).

The goal of the 360ºCHILD-profile is to provide direct access to a manageable résumé of holistic health information stored in the EMD to CHC professionals, parents, and adolescents, and to naturally guide thought processes in coherence with the chosen theoretical perspective (PHC). This dashboard supports health literacy in a way that can empower parents and adolescents to cocreate personalized plans for managing their (children’s) health, in partnership with caregivers [11,12]*.*

From the very start of the 360ºCHILD-profile’s development and research project (2012), parents, adolescents, and CHC professionals have been actively involved. Formal ideas for designing the profile’s first drafts were generated, and pilot studies showed positive reactions of stakeholders for the comprehensibility, relevance, and acceptability of the design [9]. Promising results were generated in another study related to the 360ºCHILD-profile’s reliability and validity when used by CHC medical doctors to assess child functioning [13]. In 2018, data visualization designers and researchers used their expertise and gained input from stakeholders on CHC context, usability, and user experience to redesign the 360ºCHILD-profile [9].

The current state of this newly developed 360ºCHILD-profile offers a promising, online dashboard that is ready to be introduced into CHC practice. If and how it will actually function within daily practice is yet unknown, and it is foreseen that the evaluation of effectiveness in the multidisciplinary and preventive CHC context will be complex. Therefore, a pragmatic feasibility RCT will be performed to refine our practice-derived theory on the 360ºCHILD-profile’s feasibility and potential effectiveness and to build a rationale for designing future (effect) studies (including outcome measures and sample size calculations) [14,15]. The aim of this paper is to describe the design and methods of this study that will be performed within CHC.

## Methods

### Study Design

For this pragmatic feasibility RCT, a sequential mixed methods design was chosen. First, quantitative research will be performed. Within the limitations of a feasibility study [16], an RCT will be executed with 2 parallel groups (experimental and control). Qualitative research will then be performed to understand and explain the quantitative findings and to explore the 360ºCHILD-profile’s potential benefit in CHC practice [17-19]. The study objectives will be to evaluate 2 types of feasibility: that of the 360ºCHILD-profile and that of the RCT.

- (1) The 360ºCHILD-profile feasibility evaluation will include the following: usability, including the frequency and profundity of 360ºCHILD-profile use during contacts between CHC professionals, parents, adolescents, and other caregivers; and the perspective of parents, adolescents, CHC professionals, and other caregivers on quantitative findings, requirements for the 360ºCHILD-profile’s use within CHC, and potential benefit and effectiveness.
- (2) The RCT feasibility evaluation will include the following: recruitment, retention, and response rates; acceptance of and compliance to allocated interventions; measurement completion and protocol deviations; health literacy measurement, including the variance of parent’s satisfaction on health literacy in the total and in each separate group, and a preliminary estimation of the between-group differences; the perspective of parents, adolescents, and CHC professionals on hindering and promoting factors for recruitment, retention, and response rates, acceptance and compliance to allocated intervention, measurement completion, and preliminary differences on health literacy.

### Study Population and Sample Size

The study population will mainly consist of parents and CHC professionals (nurses and medical doctors) who are involved in the care of the parent’s child (age 0-16 years) experiencing emerging problems. Adolescents (age >11 years) and other involved caregivers can also be included as participants, but children under the age of 12 years will not themselves participate. If parents or adolescents cannot comprehend the written health information (due to a language barrier or other reasons), they will not be included in this study.

For the quantitative phase, we aim to recruit 30 parents, 30 CHC professionals, 10 adolescents, and 10 other caregivers. For feasibility studies, in which outcome parameters like recruitment, response rates, and variance in the outcomes of questionnaires (SD) are measured, a sample size of 60-70 participants is justified [16,20,21]. For qualitative research with purposive sampling, it is estimated that saturation will be reached after including 20-30 participants (7-10 participants per target population; ie, parents and CHC professionals) [19,22].

### Procedure

All nurses and medical doctors working for CHC organizations in the Dutch region of South Limburg will be asked to participate. After providing informed consent, volunteers will attend a 2-hour long instructional workshop to receive information about the 360ºCHILD-profile, study procedures (inclusion and randomization), and outcome measures.

Participating CHC professionals will identify and approach eligible parents during CHC visits and eligible caregivers at the moment they become involved in the care of the parent’s children. If parents or caregivers are interested and give permission to be contacted by the researcher, researchers will start the information and informed consent procedures and enroll participants in the study after given permission.

At the end of the quantitative phase, quantitative findings will be used for purposive sampling for qualitative research to obtain a variety of perspectives from parents and caregivers and to reach a broad interpretation of the quantitative findings [19,22]. From each group within the study population (parents, adolescents, CHC professionals, and involved caregivers), 2 participants will be invited for each round of interviews. After analysis of the conducted interviews, both quantitative and qualitative findings will be used to select participants for the next round to enrich characteristics and opinions.

### Randomization and Concealed Allocation

After parents sign informed consent, they will be allocated to 1 of 2 parallel groups in a 1:1 ratio (experimental or control group) according to centralized randomization (by an independent administrator based on a protocol). The randomization plan with central block randomization (blocks of 4 and 6) will be generated beforehand by CB (not involved in the enrolment and intervention) using an online randomization system. Each phase (enrolment, randomization, quantitative outcome measurement, and analysis) will be performed independently from the others (concealed allocation), and researchers will be blinded to randomization and allocation. Parents and professionals will be, as much as possible, kept unaware of the detailed study aims related to the allocation.

### Experimental and Control Intervention

**Figure 2:**
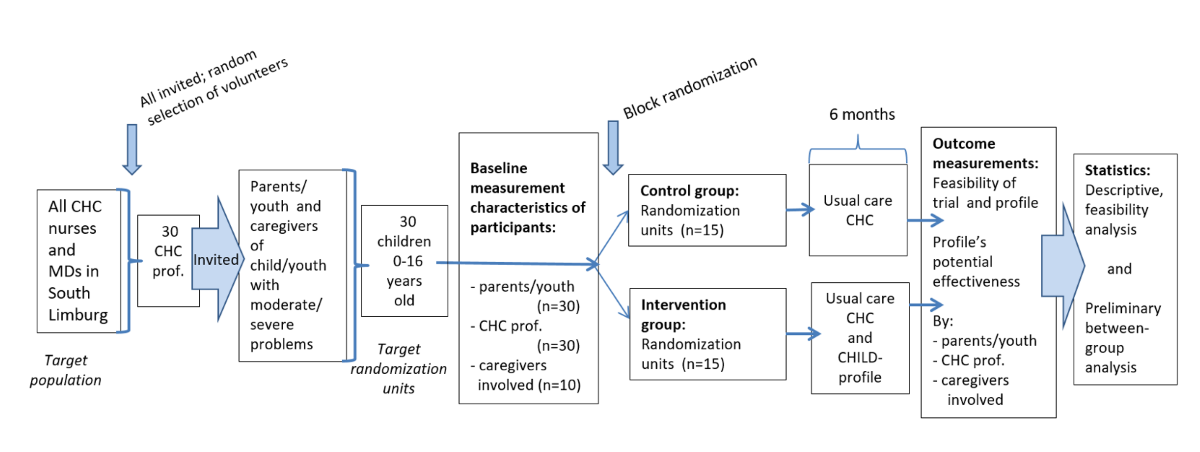
Flowchart of the randomized controlled trial's protocol. CHC: child health care; MD: medical doctor; prof: professional.

For a period of 6 months, children of the participating parents in both groups will receive usual care. Additionally, for 50% of the children (the experimental group), CHC information from the EMD will be electronically transferred to a personalized 360ºCHILD-profile. Directly after baseline measurement, the profile will be available in the EMD for CHC professionals to discuss with the parents or adolescents during the visit. After this visit, the profile also will be made accessible online for the parents or adolescents. During the 6-month follow-up period, participants will be able to consult the profile and use it to contact the caregivers whenever they want. The individual child’s health data, as presented on the profile, will not be collected and used as scientific data in the study. After the last study measurements are completed, a personalized 360ºCHILD profile will also be generated for parents or adolescents in the control group (outside the context of the study). A flowchart of the RCT’s study protocol is provided in Figure 2.

### Measures and Measurements

Questionnaires will be used to obtain baseline measurements of the following characteristics: for participating adolescents and children, information on age, gender, and level of functioning and experienced problems as indicated by CHC professionals will be measured; for parents, information on age, gender, country of birth, educational level, perspective on their child’s health and development, parenting situation, and number and age of children will be collected; for CHC professionals and other caregivers, information on discipline, educational level, experience, and perspective on the use of information technologies in health care will be collected.

An overview of the baseline measures regarding population characteristics are presented in Table 1.

**Table 1:** Baseline measures for population characteristics.

Measures	Measuring	Age group	Answer options	Reference
STEP^a^	Standardized professional’s rating of child's functioning, experienced problems, quality of environment, and needed care	0-16	5-point scale	[23]
CGAS^b^	Professional's rating of child's functioning	0-16	Continuous scale	[13,24]
PEDS^c^	Parents’ questions and concerns about child's development	0-6	3-point scale and open ended	[25]
SDQ^d^	Parents’ and adolescents’ perspective on psychological attributes	3-16	3-point scale	[26,27]
NOSIK^e^	Parents’ perspective on parenting stress^f^	2-13	6-point scale	[28]

^a^STEP: Standaard Taxatie Ernst Problematiek (only available in Dutch).

^b^CGAS: Children's Global Assessment Scale.

^c^PEDS: Parents’ Evaluation of Developmental Status.

^d^SDS: Strengths and Difficulties Questionnaire.

^e^NOSIK: Nijmeegse Ouderlijke Stress Index-Korte versie.

^f^As derived from the Parenting Stress Index-Short Form

Measurements will be conducted 6 months after baseline to measure the qualitative and quantitative outcomes of the 360ºCHILD-profile’s and RCT’s feasibility.

#### The 360ºCHILD-profile’s Feasibility

The evaluation of the 360ºCHILD-profile’s feasibility is described in this section. Quantitative outcomes on feasibility will include the following: frequency of use of the 360ºCHILD-profile by CHC professionals during contacts with parents or adolescents, as registered by CHC professionals; the profundity in which the 360ºCHILD-profiles are used during CHC visits, as indicated by CHC professionals on a questionnaire (using questions with answer options on a categorical scale).

Qualitative outcome (semistructured interviews with parents, adolescents, CHC professionals, and other caregivers) will be collected for the follow purposes: to contextualize and further inform the understanding of quantitative findings on 360ºCHILD-profile’s usability; and to explore the expectations of parents, adolescents, CHC professionals, and other caregivers regarding the 360ºCHILD-profile’s potential benefits in CHC practice.

#### RCT Feasibility

Evaluation of the RCT feasibility will occur in both group and is described in this section.

The quantitative measurements will include the following: recruitment rate (the percentages of volunteers versus invited and eligible participants); retention rate (the percentage of participants completing the study versus the participants that started); response rates (the percentages of participants per discipline who filled in and validated the questionnaire versus the number of participants who were requested); compliance to allocated intervention (the percentage of CHC professionals in the experimental group who used the 360ºCHILD-profile during a CHC visit); measure completion (the percentages of completed measures versus incomplete measures and description of missing data); protocol deviations (the description of problems encountered and eventual adaptations to the protocol for properly addressing these problems); health literacy.

Health literacy will be evaluated using the validated Dutch version of the Consumer Quality Index (CQI). The CQI is based on the Consumer Assessment of Healthcare Providers and Systems (CAHPS) and is applicable for parents of children aged 0-18 year who visit CHC [29]. The validated CQI includes questions about accessibility, ability to understand, completeness and applicability of received care, and health information and advice, with answer options on a 2-, 3-, or 4-point scale (subscale reliability: Cronbach α=.75-.82) [30]. Additionally, relevant questions from “Supplemental Items for the CAHPS on Health Information Technology” were translated from English to Dutch and incorporated into the CQI questionnaire [31]. The research team developed additional questions to ensure measurement of all dimensions of the construct’s “access to healthcare and health information” [32]. The dimensions we indicated as relevant for the CHC context and this study are availability, accommodation, accessibility, and acceptability. Affordability is not relevant in this context because CHC care is offered for free to all children and parents. This set of additional questions (n=6) with answers options on a 5-point scale will be used for the first time, and no information on the diagnostic parameters is available yet.

The qualitative measurements for RCT feasibility will include semistructured interviews with parents, adolescents, CHC professionals, and other caregivers, and will be performed for the following purposes: to contextualize and further inform the quantitative findings on recruitment, retention and response rates, compliance to allocated intervention, measurement completion, and health literacy; to explore CHC professionals’ experiences during recruitment of parents and adolescents, and participants’ satisfaction regarding their study participation and allocated intervention and perspective on requirements for a future randomized trial.

All semistructured interviews will be in person and audio-recorded. Recordings will be transcribed, and the collected data will be coded (participants will be allocated a participant number code to relate data to this code). Records will be stored in a locked place on the server separate from the other study data. Only the investigator collecting the data will have access to this documentation.

### Statistical Analysis

Baseline characteristics of all participants will be presented using descriptive statistics (mean, SD, or frequencies and range) in a table. Data of parents, adolescents, and children will be presented for the total group and for both randomized groups separately.

#### Quantitative Outcome Data

Descriptive analysis will be performed to present outcomes on the 360ºCHILD-profile’s and RCT’s feasibility (usability). The descriptive analysis of the 360ºCHILD-profile will include the following: the frequency in which the 360ºCHILD-profile is used, for which the mean and variability will be calculated; and the profundity of use of the 360ºCHILD-profile during a CHC visit, which will be presented as proportions per category.

The descriptive analysis for RCT feasibility will include the following: recruitment, retention, and response rates, compliance to allocated intervention, measurement completion, and missing data, which will be presented as logistic data and proportions; protocol deviations, which will be described using text; outcome on health literacy, including descriptions for the total sample and for each group, with continuous measures (presented as mean, SD, and CI) and categorical measures (presented as proportions).

Statistical between-group analysis will be performed to preliminarily calculate (estimates of) differences between groups (and 95% CIs), using linear mixed models. Potential confounders (for example health status, age, and education) will be evaluated and, if necessary, adjusted.

#### Qualitative Outcome Data

Analysis of each transcript will be performed by 2 independent researchers. Discrepancies will be discussed by the research team and consensus will be reached concerning codes based on expert agreement. The analysis process will include thematic analysis with open coding at the start followed by axial coding. In each phase of the analysis process, data will be reviewed using constant comparative methods. The units that will be used to describe themes and concepts can be words, sentences, or stories. Cycles of data collection and analysis will be repeated until data saturation is reached according to our research goals.

#### Connection Between Quantitative and Qualitative Results

After quantitative results are described and interpreted, they will be used to refine or adjust research questions, purposeful sampling procedures, and data collection protocols of the qualitative phase. During qualitative analysis, data will be interpreted and described separately and in coherence with quantitative results to realize the advantages of mixing both research methods, which include complementarity, triangulation, and explanation of results.

We will discuss if and how the qualitative results further the understanding or explanation of the quantitative findings and how to formulate overall conclusions and recommendations regarding theory on the 360ºCHILD-profile and the rationale for designing future (effect) studies within CHC.

## Results

The intended timeline for achieving the targeted results includes the acceptance for funding (December 2016), approval by The Medical Ethics Committee of the Maastricht University Medical Centre (no. METC azM/UM 2017-0089; July 2017), registration in the Netherlands National Register (6909; January 2018), enrolment of participants (May 2018 to September 2019), quantitative outcome data collection (March 2019 to September 2020), and qualitative data collection (October 2019 to February 2021). The results of the presented study will be available before the end of 2021. 

## Discussion

This pragmatic quantitative–qualitative study will comprehensively evaluate the feasibility of the newly developed 360ºCHILD-profile and the feasibility of conducting an RCT within the preventive setting of the CHC.

This practice-derived dashboard is new in providing a holistic and structured display (in accordance with the ICF-CY framework) of the large and complex electronic CHC data sets [13]. Earlier pilot studies, application tests, and qualitative user tests have already shown promising results for the relevance, comprehensibility, acceptability, reliability, and validity of the 360ºCHILD profile [9,13]. However, these pilots and validation tests were all performed during sessions guided by researchers in order to optimize technical and visual aspects and to increase the likelihood of usability and effectiveness. Thus, this feasibility study will generate first results on usability of this promising tool within the real-life CHC practice. This study will also generate indispensable knowledge on how to test the efficacy of this practice-derived innovation in the CHC context and is a necessary and sound intermediate step in the overall multiyear mixed methods research project [15,16].

From a pragmatic viewpoint, we searched for design options that fit current research questions and CHC context. A mixed methods design with a sequential explanatory (quantitative–qualitative) setup was chosen to enable testing our a priori, practice-based hypotheses and to give voice to parents, adolescents, and caregivers to refine theory. The pragmatic approach of the feasibility RCT enables the execution of a randomized trial within the preventive and multidisciplinary field of work and generation of results that fit this context [17]. The investment of time by CHC professionals is limited as much as possible; only a short training period is needed, each professional will need to recruit only 1-2 parent(s), and professionals are left close to daily practice during the “intervention” period (care as usual). Furthermore, CHC professionals in the experimental group do not have to drastically change their working method; they will present the personalized profile to parents and adolescents, but after that, all participants are free in choosing how (often) to use it. The between-group difference might seem rather subtle, but we expect it will have substantial impact. Our hypothesis is that the availability of the dashboard will automatically lead to efficiency (there is less wasting of time to search for data in the EMD and better quality of health literacy and early prevention of disease progression). Moreover, we expect that the theoretical structure of the profile will intuitively guide clinical reasoning in line with the context of CHC and PHC.

Information bias will be reduced by the centralized randomization, blinding of researchers for randomization, and keeping participants unaware of the detailed study aims.

Children and their parents have been chosen as the level of randomization (and not CHC professionals) to avoid bias due to differences in professionals’ working methods, characteristics, and level of experience. Contamination is avoided by virtue of the fact that, for CHC professionals in the control group, it is not possible to obtain an overview of the holistic health data from the EMD, as it is simply not available.

From the original study population, a heterogeneous population of participants will be selected for semistructured interviews to provide an in-depth insight into a broad spectrum of perspectives [33]. Interpretation of quantitative and qualitative data will be in coherence with each other, which strengthens the study’s internal validity and deepens our understanding of the findings.

This pragmatic study will ensure adequate evaluation of the currently relevant feasibility questions, and the findings will direct our decisions concerning the 360ºCHILD-profile’s implementation in Dutch CHC practice and the design of future (effect) studies. The eventual goal of this research project is to bridge the gap between the technical design of EMDs and clinical practice to enable EMDs to efficiently support CHC in its preventive tasks and give parents access to the EMD summaries. Therefore, CHC and parents can monitor health, detect deviation of normal variance and disease progression as early as possible, and cocreate preventive strategies to protect and promote children’s health—health plans that will fit each individually unique child.

## Appendix

Multimedia Appendix 1 Final decision from Dutch funding agency ZonMW.

Multimedia Appendix 2 Report of first peer-reviewer from Dutch funding agency ZonMw.

Multimedia Appendix 3 Report of second peer-reviewer from Dutch funding agency ZonMW.

Multimedia Appendix 4 Translation of peer-reviewer reports for the grant proposal and final decision from Dutch funding agency ZonMw.

## Abbreviations

CAHPS: Consumer Assessment of Healthcare Providers and Systems
CHC: child health care
CQI: Consumer Quality Index
EMD: electronic medical dossier
ICF-CY: International Classification of Functioning, Disability and Health for Children and Youth
PHC: personalized health care
RCT: randomized controlled trial
