# Supplementary material for: Evaluation of a New Personalized Health Dashboard in Preventive Child Health Care: Protocol for a Mixed Methods Feasibility Randomized Controlled Trial
Source: JMIR Res Protoc. 2021 Mar 16;10(3):e21942. doi: 10.2196/21942 (PMC8088845; doi:10.2196/21942)
Supplement: Multimedia Appendix 1 [file resprot_v10i3e21942_app1.pdf]

ZonMw stimuleert gezondheidsonderzoek en zorginnovatie

Laan van Nieuw Oost-Indië 334
2593 CE Den Haag
Postbus 93245
2509 AE Den Haag
Telefoon 070 349 51 11
Fax 070 349 51 00
www.zonmw.nl
info@zonmw.nl

Dossiernummer
70-72900-98-16108
Ons kenmerk
2016/27597/ZONMW

GGD Zuid Limburg
Gezondheidsbevordering
De heer Dr. A.M.P.M. Bovens Arts
Postbus 2022
6160 HA GELEEN

Datum
20 december 2016

Contactpersoon
Jojanneke Hillmann
Telefoon 070 349 52 67
Hillmann@zonmw.nl

Onderwerp
Honorering van uw subsidieaanvraag, projectnummer 729410001

Geachte heer Bovens,

Op 29 september 2016 heeft u bij ZonMw een subsidieaanvraag ingediend met de titel: *Doorontwikkeling van het 360°CHILDoc tot een digitaal beschikbaar kind-profiel met evaluatie van de bijdrage aan het succes van hulp binnen de zorg voor Jeugd en implementatieonderzoek* binnen de subsidieronde ‘programmalijn 4b: kennisontwikkeling over digitale innovaties die de kwaliteit van zorg in het jeugddomein verbeteren’.
Met genoegen laat ik u weten dat de werkgroep van het programma Effectief werken in de jeugdsector een positief oordeel heeft over uw aanvraag. Dat betekent dat ZonMw u de subsidie zal toekennen. In deze brief leest u hoe ZonMw tot dit oordeel is gekomen en wat u moet doen voordat uw project van start kan gaan.

Beoordeling
Uw aanvraag is beoordeeld op relevantie voor deze oproep van het programma en op kwaliteit. Voor deze ronde van het programma ontving ZonMw 20 subsidieaanvragen, waarvan er 9 wordt gehonoreerd. De beoordelingsprocedure was als volgt:

Relevantie van de aanvraag
De werkgroep van het programma Effectief werken in de jeugdsector heeft de relevantie van uw subsidieaanvraag voor deze oproep van het programma beoordeeld. Het eindoordeel over de relevantie van uw subsidieaanvraag voor het programma luidt: **relevant.**
Hierbij zijn de volgende opmerkingen van belang:

- De subsidieaanvraag voldoet aan de voorwaarden en doelstellingen van de subsidieoproep en de programmadoelstellingen.
- In de aanvraag is veel aandacht voor de doorontwikkeling van de innovatie en het toetsen van de innovatie.
- De werkgroep vindt dat u de kansen voor implementatie goed in beeld heeft. De belemmeringen zijn minder goed in kaart gebracht.
- Een punt van aandacht is de inbedding van de digitale innovatie in het bredere jeugdveld. In uw aanvraag refereert u hieraan maar dit is verder niet uitgewerkt. De werkgroep is van mening dat er nog een aantal belangrijke stappen moeten worden gezet om te komen tot bredere toepassing. Bij de uitvoering van het project dient hier aandacht voor te zijn.

Kwaliteit van de aanvraag
De werkgroep van het programma Effectief werken in de jeugdsector heeft ook een eindoordeel over de kwaliteit van uw subsidieaanvraag gegeven. Dit oordeel is gebaseerd op uw aanvraag, de beoordeling hiervan door referenten en uw reactie. Het eindoordeel over de kwaliteit van uw subsidieaanvraag luidt: **goed.**

De werkgroep geeft de volgende argumenten voor het eindoordeel:
- Met de aandachtspunten die de werkgroep u heeft meegegeven bij het positieve advies voor het uitwerken van het projectidee naar een subsidieaanvraag is over het algemeen goed rekening gehouden.
- De werkgroep vindt dat u de aanvraag goed heeft opgebouwd en dat u voor een sterk onderzoeksdesign heeft gekozen.
- Een punt van aandacht zijn de kosten van de invoering. Bij het advies over uw projectidee is meegegeven om in de uitgewerkte aanvraag duidelijk te maken hoe de digitale innovatie gebruikt kan worden binnen de bestaande (financiële) middelen. In uw aanvraag gaat u daar niet echt op in. In het projectplan bij C (pagina 16 van de subsidieaanvraag) geeft u aan dat dit in toekomstig implementatieonderzoek zal worden meegenomen: "De toekomstige implementaties zullen wederom gepaard gaan met implementatieonderzoek en na implementatie zal onderzoek worden uitgevoerd naar (kosten) effectiviteit betreffende o.a. risico inventarisatie en gezamenlijke besluitvorming en uiteindelijk de effectiviteit van hulp". Dit zou wat de werkgroep betreft iets ambitieuzer uitgewerkt mogen worden.

Op basis van beide eindoordelen heeft de werkgroep van het programma Effectief werken in de jeugdsector vervolgens een rangschikking van alle aanvragen die voor honorering in aanmerking kwamen gemaakt. Op grond hiervan heeft ZonMw uw aanvraag gehonoreerd.

We verzoeken u in het voortgangsverslag (halverwege de projectperiode) expliciet te rapporteren over:
- De mogelijke belemmeringen voor implementatie.
- Uitwerking hoe de digitale innovatie in het bredere jeugdveld ingebed wordt.
- Hoe de digitale innovatie gebruikt kan worden binnen de bestaande (financiële) middelen.

Financiering

Hoogte subsidiebedrag
De financiële bijdrage van ZonMw voor uw project bedraagt maximaal € 199.500,- voor de duur van maximaal 28 maanden. Dit bedrag is inclusief eventueel verschuldigde BTW.
Het maximale subsidiebedrag is met € 500,- lager vastgesteld, omdat de maximale subsidie voor het aanvragen van een METC verklaring € 2.000,- is en niet € 2.500,-.
Met deze toekenning ga ik eveneens akkoord met de door u/ uw partners opgevoerde uurtarieven. Hierbij wil ik uitdrukkelijk vermelden dat deze goedkeuring van uurtarieven alleen voor deze projecttoekenning geldt en dat hier voor toekomstige toekenningen geen rechten aan ontleend kunnen worden.

Subsidievoorwaarden
Zoals u weet zijn aan de financiering voorwaarden verbonden. Deze subsidievoorwaarden kunt u downloaden via de website van ZonMw:
www.zonmw.nl/subsidievoorwaarden
Ik wil u erop wijzen dat ZonMw pas een voorschot uitkeert als aan alle eisen voor het uitvoeren van het onderzoek is voldaan. Ik raad u dan ook aan eventuele procedures hiervoor tijdig te starten. Denkt u bijvoorbeeld aan een positief oordeel van een erkende medisch-ethische toetsingcommissie (METC), de Centrale Commissie Mensgebonden Onderzoek (CCMO), een projectvergunning van de Centrale

Commissie Dierproeven (CCD), of een vergunning krachtens de Wet op het Bevolkingsonderzoek (WBO). Als u niet zeker weet of uw project dergelijke verklaringen of vergunningen nodig heeft, kunt u dit nagaan bij de betreffende instanties.

Integriteit
Artikel 2, lid 3 van de Subsidiebepalingen van ZonMw impliceert dat de nationaal en internationaal aanvaarde normen van wetenschappelijk handelen worden nageleefd zoals neergelegd in de Nederlandse Gedragscode Wetenschapsbeoefening (VSNU, laatste herziene versie 31 oktober 2014), dan wel vergelijkbare codes voor niet-universitaire instellingen. In geval van (mogelijke) schending van voornoemde normen bij een door ZonMw gefinancierd project, dient ZonMw hiervan onverwijld op de hoogte te worden gesteld en dienen alle ter zake relevante documenten aan ZonMw te worden overgelegd.

ZonMw bepaalt dat de bijlage Akkoord bekostiging wetenschappelijk onderzoek 2008 en het addendum, conform artikel 7 van het akkoord niet integraal van toepassing zijn op deze subsidie. Deze worden zoveel mogelijk analoog toegepast voor zover het akkoord of het addendum niet strijdig zijn met de Algemene subsidiebepalingen van ZonMw. De Algemene subsidiebepalingen van ZonMw zijn te allen tijde leidend. Zo zal ZonMw bijvoorbeeld altijd afrekenen op basis van werkelijke kosten.

Wat moet u doen?

Belangrijk: schriftelijke bevestiging binnen vier weken
ZonMw kan u een voorschot voor het eerste projectjaar verstrekken. Dit is echter pas mogelijk als u heeft ingestemd met de subsidievoorwaarden en het project daadwerkelijk gestart is. Wilt u daarom voor **18 januari 2017** schriftelijk onderstaande informatie doorgeven aan ZonMw? Hiervoor kunt u gebruikmaken van het bijgevoegde meldingsformulier:

- uw instemming met de voorwaarden die van toepassing zijn op de toekenning van de financiële bijdrage;
- de startdatum van uw project;
- de bank- en referentiegegevens voor de betalingen van de subsidie;
- Ten aanzien van de goedkeuring van de METC of CCD:
  - Als geen verklaring(en) is vereist, stuurt u een schriftelijke bevestiging hiervan.
  - Als de verklaring(en) noodzakelijk is voor de start van het project, stuurt u de verklaring voor de start van het project aan ZonMw.
  - In het geval de verklaring(en) pas later in het project vereist is, geeft u aan wanneer de verklaring(en) nodig is. Dit is maximaal één jaar na de start van het project. ZonMw keert dan een voorschot uit voor het eerste projectjaar. Verdere voorschotten kan ZonMw alleen betalen als een kopie van de verklaring(en) is ontvangen.

Ik wijs u erop dat het project **uiterlijk 1 juni 2017** moet beginnen. Gaat het project later van start, dan vervalt de honorering van uw aanvraag. Hiervan kan alleen in zeer bijzondere gevallen worden afgeweken.

Publiekssamenvatting
ZonMw publiceert alle gehonoreerde projecten op haar website met een leesbare Nederlandse samenvatting. Deze is bedoeld voor een breed geïnteresseerd publiek met verschillende achtergronden, op taalniveau eind VWO. Zie de schrijfwijzer op http://www.zonmw.nl/nl/over-zonmw/logo-huisstijl.
Wij verzoeken u deze Nederlandse publiekssamenvatting zo spoedig mogelijk, maar tenminste binnen vier weken (vóór 18 januari 2017) na dagtekening van deze brief aan te leveren. Hiervoor kunt u in ProjectNet het tekstvak Publiekssamenvatting gebruiken (maximaal 1000 karakters, inclusief spaties).

Voortgangsverslag
ZonMw wil graag op de hoogte blijven van de voortgang van uw project. ZonMw werkt met een verkorte voortgangsrapportage die u halverwege het project indient (tenzij anders bepaald). Van het programmasecretariaat ontvangt u te zijner tijd het verzoek om een voortgangsrapportage in te dienen.
Daarnaast bent u verplicht tussentijdse wijzigingen te melden aan ZonMw. Pas na goedkeuring door ZonMw zijn de wijzigingen toegestaan.

Kennisbenutting
Resultaten van het project kunnen toepassing vinden in de praktijk, maar ook een rol spelen bij het maken van beleid, een volgende stap vormen in een wetenschappelijke carrière of de basis vormen voor een nieuw project. Om aan te geven wat er met de resultaten gebeurt, stellen wij u in voortgangs- en eindverslag diverse vragen over verspreiding- en implementatie. Ook dienen publicaties over en resultaten van het project tot vier jaar na afronding via ProjectNet aan ZonMw te worden aangeboden. Daarnaast bent u verplicht om ZonMw in deze periode te informeren over het gebruik van de resultaten.

Mocht u nog vragen hebben over deze brief, neemt u dan gerust contact op met de medewerker die in het briefhoofd vermeld staat. Als u ontevreden bent over de wijze waarop ZonMw uw aanvraag heeft behandeld, kunt u een klacht indienen (zie hieronder). Vermeld in uw communicatie met ZonMw altijd het projectnummer. Nu uw aanvraag gehonoreerd is, vervalt het oorspronkelijke nummer en geldt het nieuwe projectnummer: **729410001**.

Ik wil u nogmaals feliciteren met de honorering van uw subsidieaanvraag. Veel succes bij de uitvoering van uw project!

Met vriendelijke groet,
namens het bestuur,

i.a.

Henk J. Smid
directeur

Bijlage(n)
Meldingsformulier start project (bestemd voor hoofdaanvrager/projectleider penvoerder)

Kopie
GGD Zuid Limburg, mevrouw drs. O.H.M. van der Goot-Willms

Tegen deze beschikking kunt u bezwaar maken. In dat geval stuurt u binnen zes weken na de dag waarop het besluit bekend is gemaakt een bezwaarschrift aan het bestuur van ZonMw, t.a.v. Commissie Bezwaarschriften ZonMw, Postbus 93 245, 2509 AE Den Haag. Meer informatie over signaleren, klagen en bezwaar maken? Raadpleeg de website: www.zonmw.nl/signalerenklagenbezwaarmaken

MELDINGSFORMULIER START PROJECT ZonMw

Doorontwikkeling van het 360°CHILDoc tot een digitaal beschikbaar kind-profiel met evaluatie van de bijdrage aan het succes van hulp binnen de zorg voor Jeugd en implementatieonderzoek.

Dit formulier inzenden binnen vier weken na de dagtekening van honoreringsbrief. Graag met blauwe of zwarte pen invullen in blokletters.

1.	Projectnummer[a]	729410001 (70-72900-98-16108)
2.	Naam Projectleider	Drs. M. Weijers
3.	Toegekende subsidie	€ 199.500,-
	**START PROJECT**	
4.	Startdatum	
5.	Looptijd (in maanden)	28 maanden
6.	Einddatum	
	**BANKGEGEVENS**	
7.	IBAN<br>BIC	
8.	Ten name van:<br>te:	
9.	Bij betaling te vermelden kenmerk:	
	**CORRESPONDENTIEADRES T.B.V. FINANCIELE ZAKEN**	
10.	Naam Instelling/Afdeling	
11.	E-mailadres t.b.v. ZonMw betaalspecificaties	
12.	Adres/Postbus	
13.	Postcode/Plaats	
	**METC/CCMO/CCD/WBO**	
14.	☐ Er is geen positief oordeel van METC/CCMO[b], of een (project)vergunning van CCD/WBO[b] vereist.	
15.	☐ Er is een positief oordeel METC/CCMO[b], of een (project)vergunning van CCD/WBO[b] vereist.<br>Ik stuur een kopie van het document mee bij dit formulier.	
16.	☐ Er is in de looptijd van het project een positief oordeel van METC/CCMO[b] of een (project)vergunning CCD/WBO[b] nodig.<br>Maximaal één jaar na de start van het project stuur ik een kopie van het document op.	
	**DATAMANAGEMENT**	
17.	☐ Ik ga mijn datamanagementplan opstellen in samenwerking met:	
18.	☐ Ik ga geen nieuwe data verzamelen.	

Door ondertekening verklaart zowel de projectleider als de instelling zich akkoord met de ZonMw subsidievoorwaarden en de in toekenningsbrief opgenomen specifieke voorwaarden/uitsluitingen.

Handtekening projectleider:

Naam:

Drs. M. Weijers

Datum:

Handtekening namens bestuurlijk verantwoordelijke:

Naam: Dr. A.M.P.M. Bovens Arts

Functie:

Datum:

---

[a] Bij financiering uit additioneel VIMP-geld, hier ook het oorspronkelijke projectnummer vermelden.

[b] Doorhalen wat niet van toepassing is.
