# Supplementary material for: Evaluation of a New Personalized Health Dashboard in Preventive Child Health Care: Protocol for a Mixed Methods Feasibility Randomized Controlled Trial
Source: JMIR Res Protoc. 2021 Mar 16;10(3):e21942. doi: 10.2196/21942 (PMC8088845; doi:10.2196/21942)
Supplement: Multimedia Appendix 2 [file resprot_v10i3e21942_app2.pdf]

Subsidieprogramma / Subsidy programme	:	**Effectief werken in de jeugdsector**
Dossiernummer / Dossier number	:	**70-72900-98-16108**
Aanvrager / applicant	:	**Drs. O.H.M. van der Goot-Willms**
Projecttitel / Project title	:	**Doorontwikkeling van het 360°CHILDoc tot een digitaal beschikbaar kind-profiel met evaluatie van de bijdrage aan het succes van hulp binnen de zorg voor Jeugd en implementatieonderzoek.**
Beoordelingscode / Assessment code	:	**B.2016.01A9B**

1. Criteria

1.1 Doelstelling en vraagstelling

Denk daarbij aan:

1. Sluiten doel- en vraagstelling aan bij de doelen van de oproep?:
    a. (door)ontwikkelen en toetsen van digitale innovaties voor het jeugddomein;
    b. Inzichtelijk maken van kansen en belemmeringen voor het implementeren van deze digitale innovaties.
2. Helderheid en toetsbaarheid vraagstelling en mate van aansluiting bij de doelstelling;
3. Theoretische of empirische onderbouwing van de vraagstelling;
4. Is de doelstelling van het project helder en concreet? Is er sprake van een SMART (specifiek, meetbaar, acceptabel, realistisch en tijdgebonden) formulering?
5. Is het project innovatief? Levert het onderzoek nieuwe inzichten op? Geeft het project inzicht in de toegevoegde waarde ten opzichte van al bestaande activiteiten, producten en kennis. *(Doublures met reeds lopende of afgeronde (ZonMw) projecten komen niet in aanmerking).*

Doelstelling van het voorliggende onderzoek past bij de oproep. De ontwikkeling van een digitaal 360 CHILDdoc wat inzichtelijk is voor ouders, en waar zij belangrijke informatie aan kunnen toevoegen is is innovatief.

Het digitaal toegankelijk maken van 360 CHILDdoc wordt geëvalueerd op (tevredenheid met) de laagdrempeligheid van de JGZ en de toepasbaarheid.

Andere veronderstellingen zijn dat deze innovatie bijdraagt aan eigen regie van ouders/empowerment, bijdraagt aan een gezin, een plan en bijdraagt aan een betere gezamenlijke besluitvorming. Maar deze veronderstellingen en algemene tevredenheid worden niet getoetst in het evaluerende gedeelte van het onderzoek.

1.2 Bruikbaarheid in de uitvoeringspraktijk

Denk daarbij aan:

1. Wordt duidelijk dat de vraag voor het onderzoek afkomstig is uit de praktijk en is de relevantie voor de praktijk onderbouwd?
2. Blijkt uit de aanvraag wat de digitale innovatie inhoudt en hoe deze (naar verwachting) bijdraagt aan de psychosociale ontwikkeling van kinderen en jongeren?
3. Wordt aannemelijk dat de digitale innovatie gebruikt (gaat) word(t)(en) op het niveau van 1) de cliënt (kinderen/jongeren en/of ouders/verzorgers), 2) beroepsbeoefenaar, 3) organisatie?
4. Wordt duidelijk hoe de digitale innovatie gebruikt kan worden binnen de bestaande (financiële) middelen?
5. Is er aandacht voor de kosten van de digitale innovatie, de eigenaar ervan en de wijze waarop de innovatie onderhouden en geborgd wordt?
6. Als het om een nieuw te ontwikkelen innovatie gaat, is dit dan onderbouwd?
7. Is er afstemming met eventuele eigenaren van de digitale innovatie bij het onderzoek en is de onafhankelijkheid van het onderzoek en de resultaten gewaarborgd?

In de aanvraag wordt de relevantie voor met name ouders benadrukt. Er is aandacht voor de borging ervan in de JGZ-store, kritische knelpunten worden daarbij genoemd en hier zijn onderzoekers en organisatie alert op.
De innovatie wordt beheerd bij GGD ZL, onduidelijk is of deze ook bruikbaar blijft voor de andere praktijkinstellingen die betrokken zijn.

Een van de onderliggende aannames is dat door het digitaal toegankelijk maken van 360 CHILDdoc, ook andere zorgpartners toegang kunnen krijgen tot deze informatie (door toestemming ouders) en dat het kan bijdragen aan 1Gezin 1Plan. Dit wordt verder niet uitgewerkt in het onderzoek. Er wordt beschreven dat zorgpartners betrokken worden, maar aan welke partners gedacht wordt is niet beschreven, ook is het geen uitsluitingscriteria dat er sprake moet zijn van 1Gezin1Plan voor de evaluatie. En dit is meteen ook een knelpunt in de praktische relevantie voor andere beroepsbeoefenaren en organisaties, 360 CHILDdoc geeft informatie per kind, terwijl 1Gezin1Plan

het gehele gezin betreft. Dit knelpunt ten aanzien van praktische relevantie wordt niet benoemd in het onderzoeksvoorstel.

1.3 Plan van aanpak

Denk daarbij aan:

1. Is het plan van aanpak helder en sluit het aan op de doel- en vraagstellingen?;
2. Geschiktheid van de gekozen aanpak en methodiek. Is beargumenteerd welk type onderzoek het meest passend is om de digitale innovatie te toetsen?
3. Wordt bij het toetsen van de digitale innovatie gekeken of deze bijdraagt aan ten minste één of meerdere (bij voorkeur) van de volgende elementen:
    a. Het vergroten van de kans op succes in steun- of hulpverleningstrajecten;
    b. Het verlagen van de kosten van het steun- of hulpverleningstraject (bij gelijkblijvende kans op succes);
    c. Het verlagen van de duur van het steun- of hulpverleningstraject (bij gelijkblijvende kans op succes);
4. Wordt gekeken naar hoe de digitale innovatie geïmplementeerd wordt en wordt inzichtelijk gemaakt wat de kansen en belemmeringen voor implementatie zijn op het niveau van 1) cliënt (kinderen/ jongeren en/ of ouders/verzorgers; 2) beroepsbeoefenaar; 3) organisaties (op zowel financieel, technisch als management niveau)?
5. Wordt de doelgroep waarvoor de digitale innovatie bedoeld is, bij het project betrokken (cliënt, beroepsbeoefenaar, organisatie)?
6. Is er aandacht voor diversiteit en differentiatie van de doelgroep naar kenmerken zoals sekse, leeftijd, sociaal-economische situatie, opleidingsniveau, migratie- en culturele achtergrond en seksuele geaardheid, voor zover die relevant zijn voor de thematiek van het project?

Het plan is helder en uitgewerkt. Vooral de doorontwikkeling van 360 CHILDdoc tot online applicatie in het reeds bestaande ouderportaal is goed opgebouwd met een degelijke onderbouwde aanpak. Waarbij het ophalen van wensen, het vertalen daarvan in prototypes en weer doorontwikkelen elkaar snel opgevolgd.

Ik ben kritischer op de evaluatie van deze innovatie. Deze is uitvoerig gericht op de toepasbaarheid en het effect op de (tevredenheid met de) laagdrempeligheid van de JGZ. Terwijl er meerdere veronderstellingen onder deze tool liggen die slechts zijdelings worden getoetst. Onderzoekers geven aan dat dit empirisch nog niet mogelijk is. Maar dit betekent dat er niet wordt getoetst of de innovatie bijdraagt aan het succes van de steuntrajecten, het verlagen van de kosten of het verlagen van de duur van de ondersteuning. Het is onduidelijk of inzicht in het 360 CHILDdoc bijdraagt aan eigen regie en betere gezamenlijke besluitvorming. Het is jammer dat een van deze aannames niet diepgaander getoetst wordt. De tevredenheid met de laagdrempeligheid van de JGZ is wat smal.

Er is geen aandacht voor diversiteit en differentiatie. Het is gezien de kleine onderzoeksgroep ook de vraag of dat in deze fase van de ontwikkeling prioriteit vraagt.

1.4 Projectgroep

Denk daarbij aan:

1. Relevante expertise;
2. Vertrouwdheid met specifieke terrein;
3. Eerdere activiteiten en producten;
4. Is er sprake van een samenwerkingsverband tussen een onderzoeksinstelling en ten minste één, maar bij voorkeur meerdere, praktijkinstellingen?

Er is sprake van een breed samenwerkingsverband van meerdere praktijkinstellingen. De projectgroep heeft een brede en relevante expertise.

1.5 Haalbaarheid

Denk daarbij aan:

1. Kan met deze aanpak de doelstelling(en) worden gehaald?
2. Beschikbaarheid faciliteiten/mensen;
3. Realistische fasering en tijdplanning;
4. Geeft het projectplan een realistisch beeld van de kansen en belemmeringen voor implementatie van de digitale innovatie?

De doelstellingen kunnen behaald worden, het plan lijkt haalbaar.

2. Samenvattend Kwaliteitsoordeel

Legenda: ZG (zeer goed), G (goed), V (voldoende), M (matig), O (onvoldoende)

2.1 Samenvattend Kwaliteitsoordeel

ZG	G	V	M	O
		X		

Het voorstel is een voorstel voor een sterk en solide onderzoek naard de doorontwikkeling van 360 CHILDdoc dat digitaal toegankelijk wordt voor ouders (en jongeren) en de toepasbaarheid daarvan. Het effect van het instrument wordt enigszins smal onderzocht, en betreft slechts het effect op (de ervaren tevredenheid van) de laagdrempeligheid van de JGZ.

3. Begroting

Legenda: TH (Te hoog), R (reëel), TL (te laag)

3.1 Begroting

TH	R	TL
	X	

Gemeente en GGD ZL dragen financieel bij aan het project. Begroting is goed uitgewerkt en lijkt reëel.
