# Supplementary material for: Evaluation of a New Personalized Health Dashboard in Preventive Child Health Care: Protocol for a Mixed Methods Feasibility Randomized Controlled Trial
Source: JMIR Res Protoc. 2021 Mar 16;10(3):e21942. doi: 10.2196/21942 (PMC8088845; doi:10.2196/21942)
Supplement: Multimedia Appendix 3 [file resprot_v10i3e21942_app3.pdf]

Subsidieprogramma / Subsidy programme	:	**Effectief werken in de jeugdsector**
Dossiernummer / Dossier number	:	**70-72900-98-16108**
Aanvrager / applicant	:	**Drs. O.H.M. van der Goot-Willms**
Projecttitel / Project title	:	**Doorontwikkeling van het 360°CHILDoc tot een digitaal beschikbaar kind-profiel met evaluatie van de bijdrage aan het succes van hulp binnen de zorg voor Jeugd en implementatieonderzoek.**
Beoordelingscode / Assessment code	:	**B.2016.01A9C**

1. Criteria

1.1 Doelstelling en vraagstelling

Denk daarbij aan:

1. Sluiten doel- en vraagstelling aan bij de doelen van de oproep?:
   a. (door)ontwikkelen en toetsen van digitale innovaties voor het jeugddomein;
   b. Inzichtelijk maken van kansen en belemmeringen voor het implementeren van deze digitale innovaties.
2. Helderheid en toetsbaarheid vraagstelling en mate van aansluiting bij de doelstelling;
3. Theoretische of empirische onderbouwing van de vraagstelling;
4. Is de doelstelling van het project helder en concreet? Is er sprake van een SMART (specifiek, meetbaar, acceptabel, realistisch en tijdgebonden) formulering?
5. Is het project innovatief? Levert het onderzoek nieuwe inzichten op? Geeft het project inzicht in de toegevoegde waarde ten opzichte van al bestaande activiteiten, producten en kennis. *(Doublures met reeds lopende of afgeronde (ZonMw) projecten komen niet in aanmerking).*

Oordeel: ruim voldoende

Plus
- De aanvraag sluit aan bij de doelen van de oproep: een bestaande innovatie (360 CHILDoc) wordt doorontwikkeld en geëvalueerd waarbij kansen en belemmeringen van de innovatie worden onderzocht bij alle groepen gebruikers (JGZ, ouders, jeugdigen, zorgpartners).
- De doelstelling komt reëel en haalbaar op mij over en de geformuleerde vraagstellingen sluiten goed bij de doelstellingen aan. (Wel vind ik de huidige aanvraag wat lastig leesbaar omdat drie keer achter elkaar de doel- en vraagstelling aan bod komen, maar dan steeds een stukje gedetailleerder).
- De hele aanvraag is goed onderbouwd met (wetenschappelijke) literatuur.
- De totstandkoming en gewenstheid van de innovatie is duidelijk toegelicht (behoefte praktijk, wetenschappelijke onderbouwing, eerdere onderzoeken).

Min
- Er is minder duidelijk toegelicht hoe het 360 CHILDoc verschilt van de huidige opvraag van kindgegevens uit het DD JGZ. Wat kan de 360 CHILDoc nu allemaal precies? Zitten er naast de overzichtspagina nog meer vernieuwende aspecten in? Mijn advies zou zijn om hier nog een stuk over toe te voegen.
- Sommige termen in de doelstelling behoeven meer toelichting of uitleg hoe het geoperationaliseerd wordt, bv: context(en), tevredenheid, JGZ laagdrempeligheid.

1.2 Bruikbaarheid in de uitvoeringspraktijk

Denk daarbij aan:

1. Wordt duidelijk dat de vraag voor het onderzoek afkomstig is uit de praktijk en is de relevantie voor de praktijk onderbouwd?
2. Blijkt uit de aanvraag wat de digitale innovatie inhoudt en hoe deze (naar verwachting) bijdraagt aan de psychosociale ontwikkeling van kinderen en jongeren?
3. Wordt aannemelijk dat de digitale innovatie gebruikt (gaat) word(t)(en) op het niveau van 1) de cliënt (kinderen/jongeren en/of ouders/verzorgers), 2) beroepsbeoefenaar, 3) organisatie?
4. Wordt duidelijk hoe de digitale innovatie gebruikt kan worden binnen de bestaande (financiële) middelen?
5. Is er aandacht voor de kosten van de digitale innovatie, de eigenaar ervan en de wijze waarop de innovatie onderhouden en geborgd wordt?
6. Als het om een nieuw te ontwikkelen innovatie gaat, is dit dan onderbouwd?
7. Is er afstemming met eventuele eigenaren van de digitale innovatie bij het onderzoek en is de onafhankelijkheid van het onderzoek en de resultaten gewaarborgd?

Oordeel: ruim voldoende

Plus
- De bruikbaarheid van de innovatie in de uitvoeringspraktijk lijkt groot. Zo kan het o.a. gebruikt worden bij JGZ contactmomenten, in multidisciplinaire overlegsituaties, en is het voor ouders en jeugdigen online toegankelijk.
- Ontwikkeling van 360 CHILDoc is reeds gestart en uit eerdere onderzoeken blijkt dat het breed gedragen wordt. Met deze uitgangssituatie is doorontwikkeling kansrijk.

Min
- De in de aanvraag genoemde bijdrage van de innovatie aan epidemiologisch onderzoek is voor mij onduidelijk. Zoals ik de aanvraag lees is de innovatie vooral een visualiseringstool. Hoe draagt dit bij aan epidemiologisch onderzoek?
- Voor mij is ook niet helemaal duidelijk hoe het 360 CHILDoc verschilt van de huidige opvraag van kindgegevens uit het DD JGZ. Wat kan de 360 CHILDoc nu allemaal precies? Zitten er naast de overzichtspagina nog meer vernieuwende aspecten in? Mijn advies zou zijn om hier nog een stuk over toe te voegen.

Tot slot
Het automatisch overhevelen van gegevens uit het DD JGZ naar 360 CHILDoc lijkt me technisch behoorlijk complex. In de aanvraag staat dat in mei 2015 begonnen is met de ontwikkeling hiervan. Hoe is de stand van zaken?

1.3 Plan van aanpak

Denk daarbij aan:

1. Is het plan van aanpak helder en sluit het aan op de doel- en vraagstellingen?;
2. Geschiktheid van de gekozen aanpak en methodiek. Is beargumenteerd welk type onderzoek het meest passend is om de digitale innovatie te toetsen?
3. Wordt bij het toetsen van de digitale innovatie gekeken of deze bijdraagt aan ten minste één of meerdere (bij voorkeur) van de volgende elementen:
    a. Het vergroten van de kans op succes in steun- of hulpverleningstrajecten;
    b. Het verlagen van de kosten van het steun- of hulpverleningstraject (bij gelijkblijvende kans op succes);
    c. Het verlagen van de duur van het steun- of hulpverleningstraject (bij gelijkblijvende kans op succes);
4. Wordt gekeken naar hoe de digitale innovatie geïmplementeerd wordt en wordt inzichtelijk gemaakt wat de kansen en belemmeringen voor implementatie zijn op het niveau van 1) cliënt (kinderen/ jongeren en/ of ouders/verzorgers; 2) beroepsbeoefenaar; 3) organisaties (op zowel financieel, technisch als management niveau)?
5. Wordt de doelgroep waarvoor de digitale innovatie bedoeld is, bij het project betrokken (cliënt, beroepsbeoefenaar, organisatie)?
6. Is er aandacht voor diversiteit en differentiatie van de doelgroep naar kenmerken zoals sekse, leeftijd, sociaal-economische situatie, opleidingsniveau, migratie- en culturele achtergrond en seksuele geaardheid, voor zover die relevant zijn voor de thematiek van het project?

Oordeel: zeer goed

Plus
- De doelstelling van het project is opgedeeld in een drietal deelonderzoeken elk met een eigen vraagstelling en onderzoeksopzet. Dit maakt het overzichtelijk.

- In het eerste deelonderzoek wordt user-centered design toegepast waarbij eindgebruikers van de innovatie heel actief betrokken worden bij de ontwikkeling ervan (door hen tussenversies te laten gebruiken en hun feedback telkens in het volgende ontwerp te verwerken). Dit verhoogt de kans dat zij het ook daadwerkelijk in de praktijk zullen gaan gebruiken.
- Het tweede deelonderzoek bestaat uit een RCT met een experimentele groep (360 CHILDoc) en een controlegroep (reguliere zorg). Een RCT is een krachtige methode om het effect van een innovatie te onderzoeken.
- Alle stappen in het onderzoek zijn heel gedetailleerd uitgewerkt.

Min (potentieel)
- Sommige termen blijven onduidelijk, zoals 'context', 'JGZ laagdrempeligheid'. Wat wordt daarmee bedoeld?
- Is nagedacht over een mogelijke selectiebias van geïnteresseerde ouders. Hoe wordt gezorgd dat de mening van ouders die sceptisch zijn ook mee wordt genomen?
- Is nagedacht over privacy en geheimhouding als onderzoekers consulten bijwonen of als zorgpartners inzage krijgen in het 360 CHILDoc? Hoe wordt daarmee om gegaan?

1.4 Projectgroep

Denk daarbij aan:
1. Relevante expertise;
2. Vertrouwdheid met specifieke terrein;
3. Eerdere activiteiten en producten;
4. Is er sprake van een samenwerkingsverband tussen een onderzoeksinstelling en ten minste één, maar bij voorkeur meerdere, praktijkinstellingen?

Oordeel: goed

De projectgroep bestaat uit personen van diverse organisaties met ruime ervaring en expertise op het gebied van praktijk, onderzoek en beleid.
Sterk punt is daarnaast dat ook experts op het gebied van Interaction Design en Data Visualization Export deel uitmaken van de projectgroep.

Er mist een ondertekende samenwerkingsovereenkomst.

1.5 Haalbaarheid

Denk daarbij aan:
1. Kan met deze aanpak de doelstelling(en) worden gehaald?
2. Beschikbaarheid faciliteiten/mensen;
3. Realistische fasering en tijdplanning;
4. Geeft het projectplan een realistisch beeld van de kansen en belemmeringen voor implementatie van de digitale innovatie?

Oordeel: goed

De aanvraag komt haalbaar over, zowel qua tijdplanning als plan van aanpak en aantal projectleden.

Er wordt een evaluatie gedaan waarvan de resultaten een goed beeld zullen geven van de kansen en belemmeringen van de implementatie van de digitale innovatie.

2. Samenvattend Kwaliteitsoordeel

Legenda: ZG (zeer goed), G (goed), V (voldoende), M (matig), O (onvoldoende)

2.1 Samenvattend Kwaliteitsoordeel

ZG	G	V	M	O
	X			

Plus
- innovatie kan relevant zijn voor diverse partijen (JGZ, ouders, kinderen)
- bouwt voort op een al in gang zijnd ontwikkeltraject waarvoor al onderzoek is gedaan naar o.a. toepasbaarheid, gedragenheid, betrouwbaarheid
- plan van aanpak komt haalbaar over en heeft een duidelijke structuur. Sterk daarin zijn het user-centered design en de RCT
- projectgroep met ervaring en expertise op het gebied van praktijk, onderzoek en beleid en ook op het gebied van Interaction Design

Min
- De technische aspecten (kindgegevens uit het DD JGZ automatisch overhevelen naar 360 CHILDoc) en

privacyaspecten lijken mij complex en kunnen mogelijk een knelpunt vormen in de voortgang. Hoe is de stand van zaken?
- Er mist een ondertekende samenwerkingsovereenkomst

3. Begroting

Legenda: TH (Te hoog), R (reëel), TL (te laag)

3.1 Begroting	TH	R	TL
		X	

Door weinig ervaring met begrotingen heb ik hier geen oordeel over. Ik heb nu 'reëel' ingevuld om het formulier compleet te maken.
