# Supplementary material for: Evaluation of a New Personalized Health Dashboard in Preventive Child Health Care: Protocol for a Mixed Methods Feasibility Randomized Controlled Trial
Source: JMIR Res Protoc. 2021 Mar 16;10(3):e21942. doi: 10.2196/21942 (PMC8088845; doi:10.2196/21942)
Supplement: Multimedia Appendix 4 [file resprot_v10i3e21942_app4.docx]

**Translation of review reports and end decision of funding agency on our grant proposal:**

Regarding the Dutch review reports on the grant proposal for the ZonMw fund:

First, we shortly explain the content of the grant proposal as only part 2 is addressed in the article under review.

The grant proposal consisted of two parts:

Part 1: Development of an online 360CHILD-profile. Design process and results of part one are recently published in JMIR Formative Research: “Designing a Personalized Health Dashboard: Interdisciplinary and Participatory Approach”. <https://formative.jmir.org/2021/2/e24061>

Part 2: Evaluation of the online 360CHILD-profile. The protocol of this evaluation is presented in the article now under review.

Review reports of Grant proposal:

Review report number 1:

“OBJECTIVES: The proposal fits the objectives of the ZonMw call. The development of an online 360CHILD-profile is innovative. It will be evaluated on usability and not yet on effect regarding other intended goals of the innovation.

USABILITY IN PRACTICE: The relevance for parents is emphasized and there is attention to future implementation with attention to critical factors. The question is if the innovation will also be available for organizations outside the GGDzl (regional Community Health Services).

It is stated that the CHILD-profile will also be accessible for other care givers but this not worked out well yet. A hindering factor may be that the profile is generated for an individual child and not for a whole family and this is not mentioned in the proposal.

DESIGN: The design is clear and thorough. Especially the development of the online application in the existing parent portal has a good structure and a sound approach with cycles of gaining input, translation to prototypes etc.

Concerning the evaluation, it is directed towards usability while more goals are formulated for the innovation. These will only indirectly be evaluated as the researcher stated that this is not yet possible. Then the added value for the effectiveness of care for youth will not be evaluated.

There is not enough attention given to diversity within target population and differentiation. The question is if this is a priority in this phase of development.

PROJECT GROUP: There is a broad collaboration of more institutions and the expertise in the project group is broad and relevant.

FEASIBILITY: The goals can be met and the approach seems feasible.

QUALITY: Sufficient. Het grant proposal is strong and solid but the effect will not be profoundly evaluated yet.

BUDGET ESTIMATION: realistic. Municipality and GGDzl will contribute financially to the project. The budget estimation is thorough and seems realistic.

Review report number 2:

OBJECTIVES: More than sufficient.

*Positive remarks:*

The grant proposal does fit to the goals of the ZonMw call: an existing innovation (360CHILD-profile) will be developed further and evaluated including the chances and hindering factors of the innovation with all stakeholders (CHC, parents, youth and other care givers).

The goals are feasible and formulated research questions are consistent with the stated goals. (the readability of the grant proposal is sub optimal as goals are mentioned more than once, every time it is elaborated on in more detail.)

The whole grant proposal is thorough with good literature references.

The development so far and the need for it is clearly explained (requisite for practice, scientific background, earlier studies).

*Negative remarks:*

It is less clear what the differences are between the 360CHILD-profile and the current accessibility of data from the EMD’s. What are precisely the possibilities of the innovation? Besides the overall picture, what are other innovative aspects? My advice would be to elaborate on that a bit more.

Some terms in the objectives need more explanation on how it will be operationalised (context, satisfaction and CHC-accessibility.

USABILITY IN PRACTICE: More than sufficient.

*Positive remarks:*

The usability of the innovations in practice seems good as it can be used in consultations with parents and other caregivers and it will be online accessible for parents and youth.

The development did start earlier and pilot studies show that is broadly supported.

With this starting point, the chance for effective further development is high.

*Negative remarks:*

The added value of the innovation for epidemiologic research is not clear for me. The innovation is a tool for visualisation and how will it support epidemiologic research?

For me, it is also not totally clear how the innovation is different from the current accessibility of data from the EMD’s. What can this CHILD-profile do exactly? Are there more innovative aspects, beside the overview? My advice would be to elaborate more on that.

*Finally:* The automatic reference of CHC data from the EMD to the 360CHILD-profile seems rather complex technically. In the grant proposal is mentioned that they started in 2015 with the development of this application. What is the current status?

DESIGN: Very good.

*Positive remarks:*

The goals of the project are divided in three parts of the research with each different research questions and design. This makes it clear.

The first part of the project includes a user-centered design with active participation of end users. This enhances the chance that the innovation will actually be used in practice.

The second part of the project consists of a RCT with an experimental- and a control- group. A RCT is a powerful method to evaluate effectiveness.

All steps in the study are elaborated on in detail.

*Negative remarks:*

Some terms remain unclear, like the context, CHC-accessibility. What is meant by that?

Did researcher think about possible selection bias of interested parents? How will be assured that the opinion of parents that are more skeptical will be included?

Did researcher think about privacy matters during observations of consultations and accessibility of the CHILD-profile for other care givers? How will be dealt with these issues?

PROJECT GROUP: Good

The project group consists of persons from different organizations with experience and expertise in/on practice, research and policy making. Strong point is also that experts on Interaction Design and Data Visualization Export are part of the project group.

One of the agreements of collaborations was missing.

FEASIBILITY: Good.

The grant proposal seems feasible, regarding time schedule and approach and the number of participants in the project group.

The evaluation will provide a good picture of the chances and hindering factors for implementation of this digital innovation.

QUALITY: Good.

*Positive remarks:*

The innovation can be relevant for several stakeholders (CHC, parents, children).

The project is built on earlier work during a larger development project with research regarding applicability, the need for it and reliability.

The design seems feasible and has a clear structure with strong elements like the User-centered design and the RCT.

The project group includes experience and expertise in/on practice, research and policy making and interaction design.

*Negative remarks:*

The technical aspects (how child data are automatically referred from EMD to the CHILD-profile) and privacy issues seem complex to me and might hinder further development. What is current status on this?

BUDGET ESTIMATION**:** realistic.

I do not have much experience on this topic, so I cannot judge on the budget estimation. I did fill in “realistic” to complete the form.

End decision of Dutch funding agency ZonMw: accepted for grant, projectnumber 729410001 (after our adaptations made by researchers as and the reaction on the review reports)

The grant proposal with title: Development of the 360CHILD-profile toward an online accessible CHILD-profile and evaluation of the added value for effectiveness of Child Health Care. In grant-round 4b: development of knowledge about digital innovations for better quality of care for youth.

With pleasure, we let you know that the judgement of commission of the program Effective work within Youth Care on your grant proposal is positive; ZonMw will allocate a grant to this project. Of the in total 20 grant proposals, ZonMw allocated a grant to 9 grant proposals.

**Relevance: The end judgement is: relevant.**

The grant proposal meets conditions and goals of our program and this specific call.

The grant proposal gives much attention to the development and evaluation of the innovation.

You did envision the chances for implementations well, hindering factors to a lesser extent.

Point of interest is how it will be embedded within broad field of care for youth..

**Quality: The end judgement is: good quality.**

You did take into account the remarks made by ZonMw commission and reviewers.

The built up of the grant proposal is goed and you chose a strong research design.

Point of attentions is the costs of future implementation of the innovation.
